# Efficacy of cupping therapy in patients with the fibromyalgia syndrome-a randomised placebo controlled trial

**DOI:** 10.1038/srep37316

**Published:** 2016-11-17

**Authors:** Romy Lauche, Julia Spitzer, Barbara Schwahn, Thomas Ostermann, Kathrin Bernardy, Holger Cramer, Gustav Dobos, Jost Langhorst

**Affiliations:** 1 Department of Internal and Integrative Medicine, Kliniken Essen-Mitte, Faculty of Medicine, University of Duisburg-Essen, Essen, Germany; 2 Australian Research Centre in Complementary and Integrative Medicine (ARCCIM), University of Technology Sydney, Sydney, Australia; 3 Institute of Integrative Medicine, Department of Health, University of Witten/Herdecke, Herdecke, Germany; 4 Department of Anesthesiology, Berufsgenossenschaftliche Universitätsklinik Bergmannsheil, Bochum, Germany

## Abstract

This study aimed to test the efficacy of cupping therapy to improve symptoms and quality of life in patients diagnosed with the fibromyalgia syndrome. Participants were randomly assigned to cupping therapy, sham or usual care. Cupping was administered five times at twice weekly intervals on the upper and lower back. The primary outcome measure was pain intensity at day 18. Secondary outcomes included functional disability, quality of life, fatigue and sleep quality as well as pressure pain sensitivity, satisfaction and safety at day 18 and 6 months. Altogether 141 patients were included in this study (139 females, 55.8 ± 9.1 years). After 18 days patients reported significant less pain after cupping compared to usual care (difference −12.4; 95% CI: −18.9; −5.9, p < 0.001) but not compared to sham (difference −3.0; 95% CI: −9.9, 3.9, p = 0.396). Further effects were found for quality of life compared to usual care. Patients were mildly satisfied with cupping and sham cupping; and only minor side effects were observed. Despite cupping therapy being more effective than usual care to improve pain intensity and quality of life, effects of cupping therapy were small and comparable to those of a sham treatment, and as such cupping cannot be recommended for fibromyalgia at the current time.

The fibromyalgia syndrome is a condition characterised by chronic widespread pain in combination with fatigue, cognitive disturbances, sleep disorders and pronounced somatic and/or psychological distress^1^,^2^. Between 2.9 and 3.8% of the general population in Europe suffer from fibromyalgia^1^,^3^, the majority of them being women^2^. Only a few complementary therapies have been recommended in the treatment guidelines^4^, however cupping therapy has not been included due to the lack of evidence^5^,^6^.

Cupping therapy is an ancient medical therapy, and the common denominator in all cupping variations is the application of suction to the skin^7^,^8^. Several techniques from traditional cupping - where skin incisions are made to allow blood and other body fluids to escape - to dry cupping and cupping massage -where no such incisions are made – are available^8^. According to common theories the effects of cupping may include increased microcirculation, tissue detoxification and a subsequent relief of painful muscle tension^9^,^10^. Favourable effects of cupping therapy have been found in patients with chronic pain conditions^11^, for example neck pain^12^,^13^,^14^,^15^, low back pain^16^,^17^,^18^,^19^ or migraine^20^, however for fibromyalgia only evidence from an uncontrolled Chinese study is available showing that two weeks of daily cupping lead to a substantial decrease in pain intensity. Since patients with the fibromyalgia syndrome have reported a high prevalence for the use of complementary and alternative therapies^21^ more studies are needed to establish a robust evidence-base for the efficacy of such interventions.

This study aimed to investigate the efficacy of cupping therapy compared to usual care and a sham procedure to improve symptoms and quality of life in patients diagnosed with the fibromyalgia syndrome.

## Methods

### Ethical approval and trial registration

The trial was conducted between October 2012 and February 2015 in the Department of Complementary and Integrative Medicine in Essen, Germany. The study was approved by the ethics committee of the University Hospital Essen (approval number: 12-5056-BO) and registered at ClinicalTrials.gov (registry number: NCT01635634, date of registration 03 July 2012), prior to patient recruitment. The study was conducted according to common standard guidelines for clinical trials (Declaration of Helsinki, and the International Conference on Harmonization of Technical Requirements for Registration of Pharmaceuticals for Human Use and Good Clinical Practice (ICH-GCP) revised version, Somerset West, Republic of South Africa, 1996).

### Design

This was a randomised sham-controlled three-armed parallel group trial. The intervention group received five sessions of dry cupping, whereas the control groups received either five sessions of sham cupping, or usual care. Each group received the allocated intervention within 18 days; measurements were conducted at day 0, day 18 and after 6 months. After the measurements at day 18 usual care group patients received the cupping treatment.

### Patients

Patients were recruited via newspaper advertisements, with medical students screening interested people by phone to assess their eligibility. Patients who met the inclusion criteria were invited for assessment where they were provided with detailed written information about the study; and their written informed consent was obtained. The study physician checked patients’ medical histories, examined their physical health, and checked patients’ medical records. If patients met the inclusion criteria, and did not meet any exclusion criteria, they were included.

Trial participants were required to be between 18 and 75 years of age, and to suffer from the fibromyalgia syndrome diagnosed by a specialist. The 2010 American College of Rheumatology diagnostic criteria^22^ were checked to confirm diagnosis in all patients. Patients further had to report at least moderate pain of 45 mm or higher on a visual analogue scale (VAS)^23^, with 100 mm described as “worst pain imaginable”.

The trial exclusion criteria included pain due to inflammatory rheumatic disorder, any major psychiatric disorder, severe depression or substance abuse, any severe comorbidity such as cancer or neurological disorders, recently initiated or modified drug treatment, prior injections or acupuncture (within the past 3 months or during the trial), treatment with opioids or steroidal pain medication. Medications with non-steroidal anti-inflammatory drugs or antidepressants were admitted if the dosage was kept constant during the trial. Finally pregnant or lactating women or patients taking part in other clinical trials were excluded.

### Randomization and allocation procedure

Patients were allocated to one of three groups in sequential order adopting a computer-generated (Random Allocation Software, version 1.0.0) stratified block randomization with randomly varying block sizes. The following stratum was applied: Patients taking antidepressants vs. those without. The trial coordinator who was not involved in patients’ outcome assessments prepared sealed opaque envelopes. Envelopes were labelled according to the study participant’s identification number and after inclusion, the envelopes were opened in ascending order by the study physician and the patients were allocated to the respective intervention.

### Blinding

Patients were blinded twice: 1) to the fact that a sham procedure was used, instead they were told that two different cupping techniques would be compared against each other, i.e. traditional cupping vs. modern gentle cupping; 2) to whether they have been allocated to the real or sham cupping. Success of blinding was checked at day 18. Outcome assessors were blinded to the patients’ group allocation (all groups) at outcome assessment.

### Intervention

After the baseline measurement and randomization patients received the allocated treatment.

#### Cupping therapy

Patients in that group received five cupping treatments, two treatments per week. Cupping was performed on the patients’ upper and lower back in a semi-standardised manner. Four to eight 50–100 mm diameter acrylic glass cups were placed on the skin, and air was partially evacuated from the cups by means of a mechanical device (Pneumatron^®^ 200S, Pneumed GmbH, Idar-Oberstein, Germany). The negative pressure was adjusted to a comfortable level, and cups were fixed by means of elastic tapes. After 10 to 15 minutes cups were removed. The cups were placed on predefined locations such as the trapezius, the levator, the latissimus dorsi, or the gluteus maximus muscles; as well as individually according to the patient’s physical examination. One treatment session lasted about 30 minutes in total.

#### Sham Cupping Therapy

The same procedures were applied, however the cupping glasses had been prepared with small holes of <1 mm diameter to release the negative pressure within seconds. The cups were also fixed by means of elastic tapes. The sham treatment followed the procedure described earlier^24^. Patients in both cupping groups were told that they might feel suction initially, but this sensation would usually disappear after a few seconds, because receptors in the skin would adapt to the stretch of the skin. Patients in both cupping groups were also advised that they could continue their usual activities and therapies, but not to initiate any new therapeutic regimen for symptom management.

### Usual care group

Patients in this group were advised to continue their usual activities and therapies, but not to initiate any new therapeutic regimen for symptom management. After baseline measurement they were asked to return at day 18 for their final examination, after which they received the cupping therapy.

### Assessment

#### Patients’ Expectation

At the assessment visit all patients rated their expectations that traditional cupping or gentle cupping would improve their pain on a 100 mm VAS with 100 mm indicating ‘highest possible expectation’.

#### Compliance

The number of received cupping sessions for each patient was documented.

#### Questionnaires

Current pain intensity was measured using a 0–100 mm visual analogue scale from the German Pain Questionnaire^25^,^26^ with 0 mm indicating ‘no pain at all’ and 100 mm indicating ‘worst pain imaginable’.

The fibromyalgia impact questionnaire (FIQ)^27^,^28^ was used to determine health status and functioning of fibromyalgia patients. The FIQ is composed of 20 items, and patients can score between 0 and 100 points with higher scores indicating higher impairments. Scores above 70 usually indicate severe affliction.

Health-related quality of life was assessed using the Short Form 36 Health Survey Questionnaire (SF-36)^29^. This widely used comprehensive 36-item questionnaire yields an 8-scale health profile as well as two component summaries of physical and mental health-related quality of life.

The Multidimensional Fatigue Inventory (MFI-20) - a 20-item, multi-dimensional, self-report instrument – was used to measure severity of fatigue^30^,^31^. The instrument covers 5 dimensions; and shows good internal consistency and reliability. Possible scores for each scale range from 4–20 with higher scores indicating more severe fatigue.

Sleep disturbance was assessed on the Pittsburgh Sleep Quality Inventory (PSQI)^32^. Results of the PSQI are presented as seven subscales (max. 3 points each) and one total score (max. 21 points), with higher values indicating more severe sleep disturbances^32^.

All patients used a log to record the intensity on a 100 mm visual analogue scale, whether they took analgesics or received other treatments. For analgesics the defined daily doses were calculated^33^; for concomitant treatments the relative frequencies of days on which they received any intervention was analysed. In order to condense data and compensate for different periods of time between treatments, pain, medication and concomitant treatments between two treatments or between the last session and the measurement at day 18 were averaged.

#### Pressure pain threshold

Patients’ pressure pain thresholds (PPT) were measured using a digital algometer (Somedic AB, Hörby, Sweden) with a 1 cm^2^ probe. Pressure was applied in increments of 40 kPa/s until patients indicated a perception of pain in addition to pressure. They were measured unilaterally over the patient’s right thenar eminence for demonstration^34^; bilaterally at three predefined sites: over the levator scapulae muscle (medial to insertion on angulus superior scapulae), the descending part of the trapezius muscle (midway between C7 and the acromion process) and the gluteus maximus muscle (at the upper, outer quadrants of buttocks in anterior fold of muscle); as well as unilaterally at the site of maximal pain. The averages of three measurements each were log-transformed (lg) and analysed^35^,^36^.

#### Success of blinding

To determine whether blinding was successful patients were asked to indicate which form of cupping therapy they had received in their opinion.

#### Satisfaction with interventions

After 18 days patients were asked to judge how beneficial the respective treatment was on a 100 mm visual analogue scale with 100 mm indicating highest possible benefit, whether they would utilise this intervention in the future and whether they would recommend it to family or friends on a yes/no basis.

#### Safety

All adverse events were recorded. Patients experiencing such events were asked to see the study physician to assess their import and initiate any necessary response.

#### Primary and secondary outcome measures

The primary outcome measure was pain intensity at day 18 as measured by the visual analogue scale (VAS). Secondary outcome measures included pain (VAS), physical function (FIQ), quality of life (SF-36), fatigue (MFI), sleep quality (PSQI) at day 18 and 6 months; pressure pain sensitivity (PPT) at day 18; and pain intensity (VAS), pain medication and concomitant treatments from the daily log, compliance, satisfaction, success of blinding and safety.

### Sample size calculation

No data were available for a priori sample size calculation for cupping therapy in patients with fibromyalgia. Instead data from a reanalysis on cupping therapy were used^37^. Those data were gathered in a comparable setting with chronic neck pain patients. A minimal clinically important difference according to Lauche *et al.*^37^ was 8 mm VAS on the primary outcome with a standard deviation of 13 mm. Given the effect size of Cohen’s d = 0.615, and a two-sided 5% level t-test with a statistical power of 1-β = 80%, 43 patients would be needed to detect this group difference. We planned to include 141 patients in this trial (n = 47 per group); recognizing a potential loss of analytical power due to patient withdrawal from 10%.

### Statistical analysis

All analyses were based on the intention to treat population, i.e. each patient providing baseline data was included in the final analysis. Missing data were substituted using the arithmetical average of 50 complete data sets created by the Markov-Chain-Monte-Carlo multiple imputation procedure in SPSS. Pain and medication diary were analysed using the per-protocol population only.

The primary outcome was analysed using the univariate analysis of covariance (ANCOVA) which modelled the post-treatment outcome as a function of treatment group (classified factor), and the respective baseline value (linear covariate). A stepwise analysis was conducted; starting with the comparison cupping therapy vs. usual care; followed by cupping therapy vs. sham cupping, each with a p-value of 0.05 indicating significant differences. Within this model the treatment effect was estimated, accompanied with a 95% confidence interval. The p-value was based on a two-sided t-test within this statistical model. All other outcomes were defined as secondary outcomes and were analysed exploratively only using comparable models, without reporting p-values. This way, no alpha level adjustment was necessary to maintain the overall type I error rate of 5%^38^,^39^.

For secondary outcomes the same statistical models were used. The frequencies of responders, i.e. patients experiencing at least 30% or 50% pain reduction, were compared between groups using χ^2^ tests. Results from the daily log were analysed using a repeated measurement analysis of variance (ANOVA) with group as the between-subject-factor and pain outcomes at the different time points as the dependent variables. Medication and treatment logs were analysed with the same model. In case of significant main or interaction effects, post hoc tests (ANOVA, student’s t-tests) were conducted. All analyses were performed using the Statistical Package for Social Sciences software (IBM SPSS Statistics for Windows, release 22.0. Armonk, NY: IBM Corp.).

## Results

### Patients

**Figure 1:**
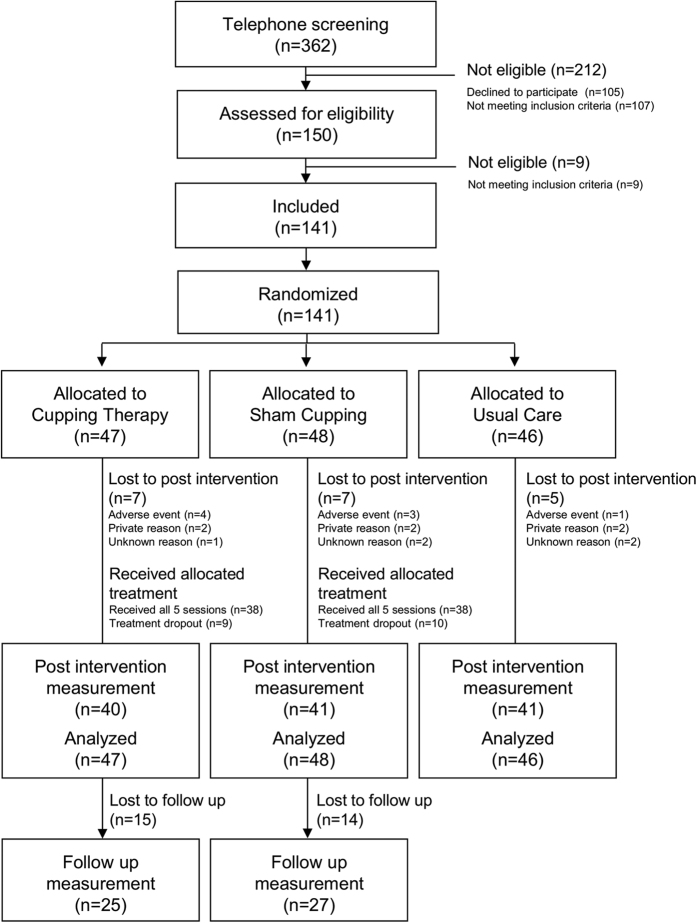
CONSORT Flowchart of patient recruitment and study flow.

From 362 patients initially screened by telephone, 150 patients were seen by the study physician, of whom 141 were enrolled. The most common reasons for excluding patients were violations of the inclusion criteria, or lost interest in the study. Of the 141 patients finally enrolled, all were randomised and allocated to their intervention. During the intervention seven patients each were lost to follow-up in cupping therapy and sham cupping, due to adverse events, private or unknown reasons. In the usual care group five patients were lost for similar reasons. A high proportion of patients withdrew from the study during the long-term follow up (see Fig. 1 for CONSORT flowchart).

### Baseline characteristics

Patients were 55.8 ± 9.1 years on average; and 139 women and two men were included, see Table 1. The majority of patients had an education below high school level, and a third each was unemployed or retired. About 40% of patients in each group were currently taking antidepressants. Patients had received many therapies in the past, mainly medication or physiotherapy. Patients reported that their pain had been present for 12 years on average.

#### Patients’ Expectation

There were no differences between the patients’ expectations towards the cupping interventions (‘traditional’ cupping: 68.1 ± 18.9 mm VAS; ‘gentle’ cupping: 64.7 ± 23.3 mm; p = 0.435).

#### Compliance

Altogether 38 out of 47 patients received five cupping treatments (80.9%), and 38 out of 48 received five sham treatments (79.2%).

### Outcome measures

#### Primary outcome measure

Analysis of pain intensity revealed a significant group difference between cupping therapy and usual care (difference −12.4, 95% CI: −18.9, −5.9, p < 0.001) at day 18 in favour of cupping, see Table 2. No group difference was found between cupping therapy and sham cupping (difference −3.0, 95% CI: −9.9, 3.9, p = 0.396), see Table 2.

After 18 days 12 patients in cupping therapy (25.5%) and nine patients in sham cupping (18.8%) reported a 30% pain reduction while only one patient in usual care did (2.2%) (p = 0.006). At least 50% pain reduction was reported by four (8.5%), five (10.4%) and zero (0.0%) patients in cupping therapy, sham cupping and usual care respectively (p = 0.091).

#### Secondary outcome measures

No difference between cupping therapy and usual care was found for physical function (FIQ) or sleep quality (PSQI). For quality of life, group differences in favour of cupping therapy were found for the scales bodily pain (difference 4.7, 95% CI: 0.9, 8.6), vitality (difference 6.3, 95% CI: 0.9, 11.7), social role functioning (difference 7.1, 95% CI: 0.1, 14.1) and mental health (difference 4.5, 95% CI: 0.0, 8.9), as well as for the mental component summary (difference 3.4, 95% CI: 0.8, 5.9), see Table 2. For fatigue only the scale reduced motivation revealed group differences (difference −1.2, 95% CI: −2.1, −0.2) in favour of cupping therapy. For pressure pain sensitivity differences were found for the left levator scapulae muscle, the left and right gluteus maximus muscle and the site of maximal pain (all p < 0.01), see Table 3.

Compared to sham cupping no differences were found for any outcomes except for one on the scale bodily pain of the SF-36 at day 18 (difference 5.1, 95% CI: 0.5, 9.6), see Table 2.

#### Success of blinding

Success of blinding was tested using χ^2^ test. Altogether 32 out of 39 (82.1%) of the patients in cupping therapy, and 30 out of 41 (73.2%) of the patients in sham cupping correctly identified their respective group allocation indicating lack of success of blinding (p < 0.0001).

#### Daily log

**Figure 2:**
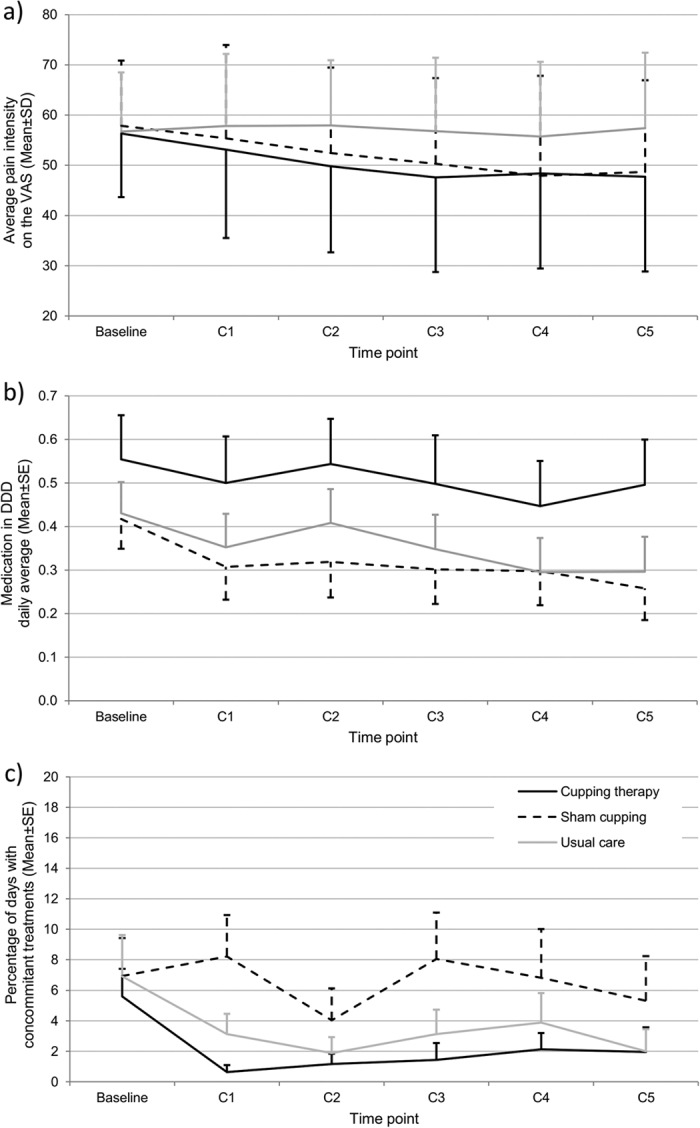
Results from the daily log, (**a**) pain intensity; (**b**) analgesic medication use and (**c**) use of concomitant therapies during the trial period, Mean and SD/SE. Please note: Each time point reflects an average of 3–4 days (C1: average of scores from the day of cupping #1 and the days until cupping #2; C2: average of scores from the day of cupping #2 and the days until cupping #3; and so forth). Averages were calculated according to individual treatment time plan. SD/SE are presented as positive or negative bars only to increase visibility.

A small but consistent decline in pain intensity was found in cupping and sham cupping, but not in usual care (Fig. 2a). Repeated measures ANOVA revealed a time × group interaction. Post hoc analysis showed a decline in pain intensity only within the cupping group. Group differences between cupping and usual care; and sham cupping and usual care were found only after the fifth cupping session. Analgesic medication (Fig. 2b) and concomitant therapies (Fig. 2c) decreased during the study period with no group differences. Altogether the rate of analgesic consumption or concomitant therapies was low in general.

#### Satisfaction with interventions

Patients were mildly satisfied with both interventions at day 18 (average benefit on a 100 mm visual analogue scale: cupping therapy: 56.9 ± 28.6 mm; sham cupping: 39.7 ± 27.2 mm). Furthermore 25 out of 39 (64.1%) would consider using cupping therapy again, so did 22 in sham cupping (57.9%). And 32 out of 39 (82.1%) and 34 out of 40 (85.0%) would recommend cupping therapy and sham cupping to family and friends respectively.

#### Safety

The following non-serious adverse events were recorded in the cupping group: two patients reported severely increased pain after cupping, one patient had an accident with bruised rips and one patient had the flu. All events resolved without intervention. A fifth patient suffered from acute torticollis which radiated into the arm but was resolved without treatment within days. In sham cupping two serious adverse events were reported: one patient with a torn meniscus and one patient with persistent pain after spinal operation. Both patients had consulted a specialist on their own. A third patient in that group had the flu. No patient in the usual care group reported adverse events.

## Discussion

This trial included 141 mainly female patients with the fibromyalgia syndrome, and found that five cupping treatments were more effective than usual care to improve pain and mental quality of life. Cupping however was not superior to sham cupping. Patients were able to distinguish the two types of cupping, and were mildly satisfied with either intervention. Adverse events related to cupping therapy mainly included increased pain, no serious adverse events related to the intervention were observed.

### Scientific evidence

Only one trial could be located during literature search^40^. Cao and colleagues investigated 30 patients. Cupping was performed daily for 15 days with bamboo cups that had been boiled in herbal decoction before the application. The authors found a pain decrease by 48% on average; and the effect was sustained at two weeks follow-up. In our current study cupping was not nearly as effective, for a number of possible reasons. Patients may not have been comparable between the studies as indicated by different pain levels and medication use. Cupping in the previous trial was also performed more frequently, and there might have been effects due to the herbal decoction. Patients in the trial were also diagnosed with the 1990 criteria of the American College of Rheumatology^2^, while diagnosis in our current trial was confirmed on the basis 2010 ACR criteria without the presence of tender point counts.

Cupping was also less effective than for chronic non-specific neck pain^12^,^13^ where a 30–50% pain reduction was found after 5 cupping treatments. However aetiology of chronic neck pain and fibromyalgia are – despite large knowledge gaps in the understanding of fibromyalgia still – rather different, with proposed abnormalities in central and peripheral pain processing in fibromyalgia patients^41^,^42^,^43^,^44^,^45^ as well as a high comorbidity of psychological disorders^46^,^47^.

Interestingly the present trial found no difference between cupping and sham cupping. This is even more surprising since the majority of patients were able to determine their respective group allocation correctly. This is in contrast to findings from Lee *et al.*^24^ who developed the sham cupping procedure. Their participants had been informed that half of them would receive sham cupping; however most participants were unsure what cupping they had received; and only a few participants uncovered their allocation. In our present trial the patients’ success to uncover the group allocation is even more remarkable because the patients were cupping naïve, i.e. they had no former experiences with cupping therapy.

One would think that once the patients uncover their group assignment the sham intervention would be inefficacious. But it was never revealed to the participants that a sham intervention was used, it was introduced as ‘gentle’ cupping instead. This is in line with findings from a systematic review that found the magnitude of the placebo effect larger in trials with physical placebos and trials where the use of a placebo interventions were not disclosed^48^. The same review did not find significant effects of placebo interventions in pain conditions, however results of included trials showed inconsistent effects of placebo effects^48^. The gentle therapy was indeed preferred over the traditional cupping therapy and explicitly requested by trial participants. Therefore factors such as expectations^49^, patient-practitioner-interaction and other positive experiences with complementary therapies may have added to this effect. Furthermore even the sham procedure included some minor stimulation in the beginning, when the cups were placed on the back, and the air was evacuated from the cups. Therefore the sham may be considered a minimal cupping, and exert effects similar to those that have been observed in trials using sham acupuncture for low back pain^50^,^51^.

### Strengths and limitations

The strengths of the study include the randomised study design; the use of two different comparators; and the blinding of patients in the active treatment groups as well as outcome assessors. Outcomes were further chosen according to the guiding symptoms of fibromyalgia according to current definitions^4^,^52^; however only pain was selected as the primary outcome because it was considered the most important outcome. The low number of drop-outs at the post-intervention measurement and the overall good compliance also indicated that the tested interventions were tolerable. Finally analgesic medication and concomitant treatments were evaluated and their influence on the outcomes ruled out.

Limitations include the non-successful blinding of patients, however due to the positive connotation of the seemingly gentle cupping the placebo effect was preserved. Patients in the study were mainly female in their mid-40 and 50’s, limiting the significance of the trial’s findings for male, younger and older patients with fibromyalgia. It is known that the vast majority of fibromyalgia patients are female with only 1 in 10 patients being male^2^. The inclusion of two male patients only might be related to different factors, including the female patients’ preference towards complementary and alternative medicine, however similarly low rates of males have been found in many other studies including CAM^53^, pharmacotherapy^54^ studies or psychotherapy^55^. Further studies should examine the barriers for male fibromyalgia patients to participate in clinical trials, and ensure that more male patients are approached directly to increase a balanced sample.

### Future studies

More studies are warranted to evaluate the effects of different cupping techniques of different durations on chronic pain syndromes. For fibromyalgia in particular the effects in patients with certain subtypes should be determined. It may also be interesting to test cupping as part of a multimodal treatment approach, or in combination with therapies like acupuncture. Further studies should also re-evaluate the sham cupping device and – if necessary – develop reliable alternatives.

## Conclusion

Five cupping treatments were more effective than usual care to improve pain intensity and quality of life in patients diagnosed with the fibromyalgia syndrome. Given that effects were small, and cupping was not superior to sham cupping treatments currently no recommendation for cupping in the treatment of fibromyalgia can be made. Further research is warranted for conclusive judgement of the efficacy of cupping therapy for chronic pain.

## Additional Information

**How to cite this article**: Lauche, R. *et al.* Efficacy of cupping therapy in patients with the fibromyalgia syndrome-a randomised placebo controlled trial. *Sci. Rep.* **6**, 37316; doi: 10.1038/srep37316 (2016).

**Publisher’s note**: Springer Nature remains neutral with regard to jurisdictional claims in published maps and institutional affiliations.

## Figures and Tables

**Table 1:** Baseline characteristics of trial patients according to study arms: Cupping therapy, sham cupping and usual care.

Item	Cupping Therapy N = 47	Sham Cupping N = 48	Usual Care N = 46
Age in years, Mean ± SD	54.35 ± 10.6	56.3 ± 8.7	56.8 ± 7.7
Gender n (female)/n (male)	46/1	47/1	46/0
BMI in kg/m^2^, Mean ± SD	29.4 ± 7.3	27.2 ± 4.7	28.2 ± 5.4
*Marital status*, *n* (%)			
Single	5 (10.6)	2 (4.2)	4 (8.7)
In relationship, married	31 (66.0)	32 (66.7)	30 (65.2)
Separated, divorced, widowed	11 (23.4)	14 (29.2)	12 (26.1)
*Education*, *n* (%)			
<High school	35 (74.5)	33 (68.8)	32 (69.6)
High school	10 (21.3)	8 (16.7)	6 (13.0)
University degree	2 (4.3)	7 (14.6)	8 (17.4)
*Employment*, *n* (%)			
Unemployed	14 (29.8)	12 (25.0)	4 (8.7)
Employed	16 (34.0)	19 (39.6)	21 (45.7)
Retired (health related)	17 (11)	17 (9)	21 (16)
Duration of pain in years, Mean ± SD	11.6 ± 9.2	11.2 ± 8.9	13.0 ± 8.4
*Previous therapies*, *received*, *n* (%)			
Medication	38 (80.9)	42 (87.5)	38 (82.6)
Physiotherapy	36 (76.6)	38 (79.2)	35 (76.1)
Chiropractic	13 (27.7)	14 (29.2)	14 (30.4)
Psychotherapy	22 (46.8)	29 (60.4)	20 (43.5)
Acupuncture	29 (61.7)	33 (68.8)	32 (69.6)
Relaxation	26 (55.3)	25 (52.1)	19 (41.3)
Rehabilitation center	27 (57.5)	26 (54.2)	25 (54.4)
*Current Medication*, *n* (%)			
Antidepressants	18 (38.3)	18 (37.5)	17 (37.0)
*Expectation (Mean* ± *SD*)			
Traditional Cupping, mm VAS	68.1 ± 18.9	67.5 ± 20.8	73.4 ± 19.2
“Gentle Cupping” (Sham), mm VAS	70.6 ± 21.4	64.7 ± 23.3	71.4 ± 21.8

**Table 2:** Results of statistical analysis for pain and quality of life between trial groups at day 18 and 6 months.

	Cupping			Usual Care		Estimated group difference between Cupping and Usual Care at Day 18	Sham			Estimated groupdifference between Cupping and Sham at Day 18	Estimated group difference between Cupping and Sham at 6 Months
	Baseline	Day 18	6 Months	Baseline	Day 18		Baseline	Day 18	6 Months		
Pain intensity (mm VAS)	55.5 ± 21.0	51.1 ± 20.9	58.5 ± 17.5	54.5 ± 20.9	62.9 ± 18.7	−12.4 (−18.9; −5.9) p < 0.001	58.9 ± 22.3	56.4 ± 23.91	60.7 ± 17.8	−3.0 (−9.9; 3.9) p = 0.396	−1.2 (−8.0;5.5)
*Function (FIQ*)											
FIQ total score	55.7 ± 12.8	53.2 ± 16.8	56.9 ± 14.9	61.4 ± 14.1	61.5 ± 15.4	−2.5 (−6.0; 1.1)	61.8 ± 11.8	58.3 ± 13.4	61.2 ± 14.1	0.5 (−3.7; 4.8)	0.4 (−4.2; 5.0)
*Quality of life (SF-36*)											
Physical component summary	31.6 ± 7.3	34.0 ± 8.6	34.3 ± 8.4	31.2 ± 7.6	32.9 ± 7.3	0.8 (−1.3; 2.9)	31.7 ± 7.4	32.4 ± 8.5	32.7 ± 8.8	1.8 (−0.6; 4.1)	1.7 (−0.9; 4.3)
Mental component summary	41.1 ± 12.1	43.9 ± 11.8	39.9 ± 12.4	40.1 ± 13.0	39.6 ± 13.8	3.4 (0.8; 5.9)	37.6 ± 11.1	40.2 ± 11.2	37.2 ± 11.6	0.9 (−2.2; 3.9)	−0.1 (−3.4; 3.3)
Physical functioning	50.3 ± 24.5	53.4 ± 22.2	55.7 ± 20.2	48.9 ± 21.4	48.2 ± 19.0	4.2 (−0.7; 9.1)	48.1 ± 20.8	49.1 ± 20.0	51.2 ± 20.4	2.7 (−2.5; 7.8)	3.2 (−3.1; 9.4)
Physical role functioning	23.8 ± 27.3	39.5 ± 36.3	35.5 ± 29.4	19.6 ± 29.8	30.2 ± 37.0	5.8 (−5.8; 17.5)	11.5 ± 22.5	23.3 ± 30.6	25.1 ± 25.6	7.0 (−4.8; 18.8)	3.2 (−6.7; 13.1)
Bodily Pain	29.1 ± 13.9	38.5 ± 16.1	33.5 ± 14.0	26.5 ± 14.1	31.5 ± 14.1	4.7 (0.9; 8.6)	29.0 ± 9.9	33.4 ± 14.3	30.8 ± 14.4	5.1 (0.5; 9.6)	2.7 (−2.6; 7.9)
General Health Perception	39.2 ± 16.1	42.4 ± 18.6	42.2 ± 20.2	41.5 ± 18.0	43.9 ± 16.6	0.4 (−3.9; 4.7)	42.6 ± 14.6	42.2 ± 16.5	39.9 ± 15.5	2.9 (−2.2; 8.0)	5.1 (−0.1; 10.4)
Vitality	33.5 ± 18.6	38.9 ± 19.9	34.6 ± 19.2	31.3 ± 17.8	30.7 ± 21.2	6.3 (0.9; 11.7)	31.2 ± 16.6	33.2 ± 14.9	31.0 ± 14.0	4.1 (−1.4; 9.7)	2.2 (3.3; 7.8)
Social role functioning	55.3 ± 27.6	60.6 ± 26.3	56.7 ± 24.4	52.6 ± 27.9	51.5 ± 28.3	7.1 (0.1; 14.1)	48.9 ± 19.1	54.6 ± 23.0	52.2 ± 23.4	0.7 (−6.7; 8.2)	−0.9 (−7.8; 5.9)
Emotional role functioning	43.2 ± 42.8	56.0 ± 42.7	45.0 ± 33.6	48.6 ± 45.9	48.5 ± 41.9	10.8 (−2.4; 24.1)	31.9 ± 39.5	45.0 ± 41.2	39.7 ± 34.3	2.8 (−9.3; 14.9)	−0.1 (−11.6;11.5)
Mental health	56.3 ± 18.1	60.5 ± 19.4	55.1 ± 19.7	50.6 ± 19.7	50.4 ± 23.4	4.5 (0.0; 8.9)	50.3 ± 16.7	53.2 ± 18.0	48.9 ± 18.1	2.4 (−2.5; 7.3)	1.2 (−3.9; 6.2)

Estimated group differences from the ANCOVA and 95% Confidence Intervals (CI) are presented.

**Table 3:** Results of statistical analysis for secondary outcomes between trial groups at day 18 and 6 months.

	Cupping			Usual Care		Estimated group difference between Cupping and Usual Care at Day 18	Sham			Estimated group difference between Cupping and Sham at Day 18	Estimated group difference between Cupping and Sham at 6 months
	Baseline	Day 18	6 months	Baseline	Day 18		Baseline	Day 18	6 months		
*Pain perception (SBL*)											
Affective pain perception	4.2 ± 3.7	4.6 ± 3.7	4.8 ± 3.3	5.0 ± 3.4	5.3 ± 3.2	0.1 (−0.9; 1.2)	4.3 ± 3.8	4.1 ± 3.3	4.9 ± 2.8	0.6 (−0.6; 1.7)	−0.0 (1.0; −1.0)
Sensory pain perception	11.6 ± 4.4	11.8 ± 4.4	12.5 ± 4.2	9.9 ± 5.8	11.3 ± 5.2	−0.6 (−2.2; 1.0)	11.6 ± 6.0	12.0 ± 5.2	13.1 ± 5.4	0.2 (−1.7; 1.2)	−0.5 (−2.3; 1.3)
*Fatigue (MFI*)											
General fatigue	15.2 ± 3.5	14.9 ± 3.7	14.7 ± 3.1	16.0 ± 3.3	15.9 ± 3.3	−0.3 (−1.3; 0.6)	15.6 ± 3.1	15.1 ± 2.9	15.3 ± 2.8	0.1 (−1.0; 1.1)	−0.5 (−1.6; 0.7)
Physical fatigue	14.6 ± 2.7	14.4 ± 3.5	14.6 ± 2.6	15.2 ± 3.4	14.9 ± 3.4	−0.1 (−1.2; 1.0)	14.8 ± 2.5	14.1 ± 2.6	14.9 ± 3.0	0.3 (−0.8; 1.3)	−0.2 (−1.3; 0.9)
Reduced motivation	11.2 ± 3.2	11.0 ± 3.1	11.6 ± 3.1	11.2 ± 3.8	12.2 ± 3.5	−1.2 (−2.1; −0.2)	11.1 ± 3.2	11.3 ± 3.1	11.4 ± 2.9	−0.3 (−1.3; 0.8)	0.2 (−1.0; 1.3)
Reduced activity	12.4 ± 2.7	13.4 ± 3.5	13.5 ± 2.9	13.7 ± 3.6	14.0 ± 3.8	−0.7 (−2.3; 0.8)	13.2 ± 3.9	13.2 ± 2.8	14.1 ± 3.0	0.1 (−1.2; 1.5)	−0.7 (−1.9; 0.5)
Mental fatigue	12.7 ± 3.9	12.6 ± 4.1	13.2 ± 3.5	13.2 ± 4.3	13.2 ± 3.8	−0.3 (−1.3; 0.7)	13.7 ± 2.7	13.4 ± 3.2	13.5 ± 3.3	−0.0 (−0.9; 0.9)	0.3 (−0.8; 1.4)
*Sleep (PSQI*)											
Subjective sleep quality	1.7 ± 0.7	1.6 ± 0.7	1.7 ± 0.6	2.1 ± 0.7	1.9 ± 0.7	−0.1 (−0.3; 0.1)	1.9 ± 0.8	1.8 ± 0.8	1.9 ± 0.6	−0.1 (−0.3; 0.1)	−0.1 (−0.3; 0.1)
Sleep latency	1.7 ± 0.9	1.4 ± 1.0	1.6 ± 0.8	1.5 ± 1.0	1.7 ± 1.0	−0.3 (−0.7; 0.1)	1.8 ± 0.8	1.7 ± 0.8	1.7 ± 0.7	−0.2 (− 0.6; 0.1)	−0.0 (0.3; −0.3)
Sleep duration	1.5 ± 1.2	1.6 ± 1.1	1.6 ± 0.9	1.5 ± 1.1	1.6 ± 1.0	0.0 (−0.4; 0.4)	1.7 ± 1.1	1.8 ± 1.1	1.7 ± 0.9	−0.2 (− 0.6; 0.3)	−0.1 (0.5; 0.2)
Habitual sleep efficiency	1.3 ± 1.2	1.6 ± 1.2	1.6 ± 0.9	1.4 ± 1.2	1.5 ± 1.1	0.1 (− 0.4; 0.6)	1.5 ± 1.2	1.8 ± 1.1	1.7 ± 1.0	−0.3 (−0.7; 0.2)	−0.1 (−0.5; 0.3)
Sleep disturbances	1.8 ± 0.7	1.7 ± 0.6	1.8 ± 0.5	1.7 ± 0.7	2.0 ± 0.7	−0.2 (−0.5; −0.0)	1.8 ± 0.6	1.9 ± 0.7	1.8 ± 0.6	−0.2 (−0.4; 0.1)	−0.0 (−0.3; 0.2)
Use of sleep medication	0.8 ± 1.2	0.7 ± 1.0	0.8 ± 0.9	0.5 ± 1.0	0.7 ± 1.0	−0.2 (−0.5; 0.1)	0.3 ± 0.8	0.6 ± 0.9	0.4 ± 0.5	−0.2 (−0.5; 0.1)	0.2 (−0.1; 0.5)
Daytime dysfunction	1.7 ± 0.8	1.7 ± 0.8	1.9 ± 0.7	1.8 ± 0.8	2.1 ± 0.8	−0.3 (−0.5; 0.0)	2.0 ± 0.7	1.8 ± 0.6	1.9 ± 0.6	0.1 (−0.2; 0.3)	0.1 (−0.2; 0.3)
Global PSQI Score	10.3 ± 3.9	10.0 ± 4.0	10.8 ± 3.5	10.8 ± 3.8	11.2 ± 3.6	−0.8 (−1.7; 0.1)	10.8 ± 4.0	11.3 ± 4.3	10.9 ± 3.8	0.9 (−0.1; 1.8)	0.3 (−0.7; 1.2)
*Pressure pain sensitivity*											
Thenar	2.2 ± 0.2	2.1 ± 0.2		2.2 ± 0.2	2.1 ± 0.3	0.0 (0.0; 0.1)	2.1 ± 0.2	2.1 ± 0.2		−0.0 (−0.1; 0.0)	
Left Trapezius	2.0 ± 0.2	2.1 ± 0.2		2.1 ± 0.2	2.0 ± 0.2	0.1 (0.0; 0.1)	2.0 ± 0.2	2.0 ± 0.2		−0.0 (0.0; 0.1)	
Right Trapezius	2.0 ± 0.3	2.0 ± 0.2		2.0 ± 0.2	2.0 ± 0.3	0.1 (0.0; 0.1)	2.0 ± 0.3	2.0 ± 0.2		−0.0 (0.1; −0.1)	
Left Levator	2.0 ± 0.2	2.1 ± 0.2		2.1 ± 0.3	2.0 ± 0.3	0.1 (0.0; 0.1)	2.0 ± 0.2	2.0 ± 0.2		−0.0 (0.1; −0.1)	
Right Levator	2.0 ± 0.3	2.1 ± 0.2		2.1 ± 0.3	2.0 ± 0.3	0.1 (0.0; 0.1)	2.0 ± 0.3	2.0 ± 0.2		−0.0 (−0.1; 0.0)	
Left Gluteus	2.0 ± 0.2	2.1 ± 0.2		2.1 ± 0.2	2.0 ± 0.3	0.1 (0.0; 0.1)	2.0 ± 0.2	2.1 ± 0.3		−0.0 (0.1; −0.1)	
Right Gluteus	2.0 ± 0.3	2.1 ± 0.2		2.1 ± 0.2	2.0 ± 0.3	0.1 (0.0; 0.2)	2.0 ± 0.3	2.1 ± 0.2		−0.0 (0.1; −0.1)	
Site of maximal pain	1.9 ± 0.2	2.0 ± 0.2		2.0 ± 0.2	1.9 ± 0.3	0.1 (0.0; 0.2)	1.9 ± 0.2	1.9 ± 0.3		−0.0 (0.1; −0.1)	

Estimated group differences from the ANCOVA and 95% Confidence Intervals (CI) are presented.
